# The potential impacts of community drug checking within the overdose crisis: qualitative study exploring the perspective of prospective service users

**DOI:** 10.1186/s12889-021-11243-4

**Published:** 2021-06-16

**Authors:** Bruce Wallace, Thea van Roode, Flora Pagan, Dennis Hore, Bernadette Pauly

**Affiliations:** 1 Canadian Institute for Substance Use Research, University of Victoria, PO Box 1700, STN CSC, Victoria, BC Canada; 2 School of Social Work, University of Victoria, PO Box 1700, STN CSC, Victoria, BC Canada; 3 Department of Chemistry, University of Victoria, PO Box 1700, STN CSC, Victoria, BC Canada; 4 Department of Computer Science, University of Victoria, PO Box 1700, STN CSC, Victoria, BC Canada; 5 School of Nursing, University of Victoria, PO Box 1700, STN CSC, Victoria, BC Canada

**Keywords:** Drug checking, Harm reduction, Substance use, Fentanyl

## Abstract

**Background:**

As drug checking becomes more integrated within public health responses to the overdose crisis, and potentially more institutionalized, there is value in critically questioning the impacts of drug checking as a harm reduction response.

**Methods:**

As part of a pilot project to implement community drug checking in Victoria, BC, Canada, in-depth interviews (*N* = 27) were held with people who use or have used substances, family or friends of people who use substances, and/or people who make or distribute substances. Critical harm reduction and social justice perspectives and a socioecological model guided our analysis to understand the potential role of drug checking within the overdose crisis, from the perspective of prospective service users.

**Results:**

Participants provided insight into who might benefit from community drug checking and potential benefits. They indicated drug checking addresses a “shared need” that could benefit people who use substances, people who care for people who use substances, and people who sell substances. Using a socioecological model, we identified four overarching themes corresponding to benefits at each level: “drug checking to improve health and wellbeing of people who use substances”, “drug checking to increase quality control in an unregulated market”, “drug checking to create healthier environments”, and “drug checking to mediate policies around substance use”.

**Conclusions:**

Drug checking requires a universal approach to meet the needs of diverse populations who use substances, and must not be focused on abstinence based outcomes. As a harm reduction response, community drug checking has potential impacts beyond the individual level. These include increasing power and accountability within the illicit drug market, improving the health of communities, supporting safer supply initiatives and regulation of substances, and mitigating harms of criminalization. Evaluation of drug checking should consider potential impacts that extend beyond individual behaviour change and recognize lived realities and structural conditions.

**Supplementary Information:**

The online version contains supplementary material available at 10.1186/s12889-021-11243-4.

## Background

The province of British Columbia (BC) is considered the epicenter of the overdose crisis in Canada. In 2016, the Province’s Health Officer declared illicit drug overdoses a public health emergency [1, 2], with rates of overdose fatalities exceeding 30 per 100,000 individuals [3] and illicit fentanyl detected in 87% drug overdose deaths [4]. Since the declaration of the public health emergency, drug checking has been pursued as part of an integrated harm reduction response in the province [5].

As drug checking becomes more integrated within public health responses to the overdose crisis, and potentially more institutionalized, there is value in critically questioning the impacts of drug checking as a harm reduction response within this context [6]. Groves (2018) [7] argues that most evaluations of drug checking have focused on individual behaviour change, asking “what would people do?” Here, effectiveness is often measured in abstinence terms such as individuals’ disposal or non-use of their tested substances. He argues that a more pragmatic approach to drug checking is required, stating “abstinence is a goal that displays ignorance of reality”. Indeed, disposal or non-use of substances may not be a goal or aim for many who use substances. Privileging these particular ends ignores both the lived realities of people who use substances within the context of criminalization, and the value of personal harm reduction strategies [8]. Furthermore, drug checking technologies are developing rapidly and are able to provide increasingly more information than before. Thus, it is critical to better understand the potential role of community-wide drug checking within this context of inequities, stigmatization and criminalization and rapidly changing technologies.

With critics claiming that drug checking could condone drug use (specifically at music festivals) [9], evaluations have often focused on the potential for drug checking to influence individual’s drug-taking behaviour, and measure disposal or non-use of substances as a means of increasing acceptance [10]. For example, an early study of regular ecstasy users [11] sought to counter critics by stating their research indicated that pill testing could in fact reduce use. More recently, a study of people who attend festivals in Portugal [12] measured non-use, with 94% of participants reporting they would not take a drug when test results confirmed an unexpected substance. A survey of people who attend festivals in Australia [13] asked young people if they would or would not take their substance if test results confirmed the presence of additional ingredients. The majority reported they would not take the substance, supporting the role of drug checking in influencing their drug use. Similarly, research from a festival in Canada [14] documents people discarding their substances as a positive indicator, as discarding increased when test results differed from the expected substance. These studies focus on individual behaviour change within a limited context. However, Measham [15] notes the potential for drug checking to inform on use of the local drug market, highlighting that drug checking may have benefits that extend beyond this context or these measures of individual behaviour change.

Currently, drug checking is undergoing significant shifts with an exponential increase in new services, instruments and analytical techniques [10, 16–20]. These newer drug checking technologies are increasingly being included in public health responses to the current overdose epidemic, where fentanyl and other potent ingredients are linked to unprecedented numbers of overdose deaths [18]. The emerging responses are raising new questions [19]: Will people who inject alone, and face significant risk of overdose, access drug checking services [21]? What are the factors that influence people to access community drug checking, and how can emerging services be responsive to structural vulnerabilities [12, 22, 23]? How can people who sell substances, access and benefit from drug checking technologies and provide more accurate information to consumers [24]? And overall, how will different models of community drug checking respond to differing contexts and needs [10]?

The above questions illustrate the complex and varied context and populations for community-wide drug checking, where the role and impacts of drug checking have not been well explored. Community drug checking faces significant challenges within a societal context of criminalization, stigmatization and violence and it is critical to attend to these structural conditions [8]. This has implications for how drug checking services are used and the associated impacts. While there are obvious benefits to responding to drug use as a health issue rather than a criminal one, the emphasis on health continues to focus on individual behaviour change rather than social determinants and inequities [25]. Furthermore, harm reductionists have cautioned that basic harm reduction principles can be lost in implementation of programs, and that engagement of people who use substances is critical for services to be inclusive and responsive [6]. Moreover, harm reduction alone is inadequate as these responses typically consider individuals and their substance use, risk behaviours and harms as isolated from social contexts including inequities, stigmatization and criminalization [26]. Overall, when harm reduction responses are solely focused on reducing the harms of drug use to the individual, the social systems that contribute and sustain harms are ignored and by default perpetuated [26].

A critical harm reduction and social justice perspective on drug checking includes a more comprehensive approach that considers impacts on multiple levels, from the individual to the policy and systems levels, within this context of inequities, stigmatization and criminalization. Our research seeks to examine the potential impacts of community drug checking from the perspective of prospective service users. In this study, we explore how community drug checking may have different impacts beyond individual behaviour change, when examined through a socioecological model. We drew on critical perspectives of harm reduction and social justice to reconceptualise effectiveness of drug checking services within the context of an illicit drug overdose crisis, and the ongoing criminalization and stigmatization of people who use substances.

## Methods

This study draws from community-based qualitative research conducted as part of a pilot project to implement and operate community drug checking in Victoria, BC, Canada. Victoria is the capital of BC, and one of the areas in the province most impacted by overdose deaths. The pilot project was initiated by Wallace (PhD, he/him) in Social Work and Hore (PhD, he/him) in Chemistry and Computer Science, and this qualitative inquiry was undertaken to inform implementation of this pilot project. Wallace, Pauly (RN, PhD, she/her) and Pagan (MSW, she/her) have long standing relationships with local harm reduction groups including the drug user organization, as well as the regional health authority, and van Roode (PhD, she/her) is a Research Associate working on both the pilot project and the qualitative research.

We collected data from November 2018 to February 2019. In-depth qualitative interviews (*N* = 27) were conducted with people who use or have used substances, family or friends of people who use substances, and/or people who make or distribute substances. Recruitment of research participants occurred at the harm reduction sites that were proposed as locations for the pilot project’s drug checking services. This included a drug user organization that provides services by and for people who use substances; a community harm reduction site that includes a needle exchange, a drop-in medical clinic, as well as other services; and a suburban harm reduction site that offers opioid substitution therapy, along with other services.

A qualitative interview guide was developed by BW, TvR, and FP, with input from BP and DH, to seek perspectives on how best to deliver drug checking services within the context of the overdose crisis, and potential impacts of drug checking services (Additional File 1). This was pilot tested and revised with community collaborators to be relevant, respectful and recognize the structural impacts of stigma and criminalization. Pilot interviews were facilitated by a local drug user organization. The staff at the harm reduction sites supported participant recruitment by distributing recruitment handbills and emails to share with people accessing the sites. The sampling was predominantly convenience sampling as we interviewed those individuals who expressed interest. We did purposefully schedule data collection to reach people accessing services at the different sites. Of the 27 interviews, we conducted 14 interviews at the local drug user organization, 7 interviews at the community harm reduction site, and 6 interviews at the suburban harm reduction site. Interviews were ended as we were noticing frequent redundancies in the interviews as well as less new interview possibilities at the recruitment sites. While there is certainly much more information to collect, we concluded that we had likely saturated the potential of our approved recruitment sites.

Interviews were conducted by either a community or university researcher (BW & FP) and audio-recorded and transcribed verbatim. Interviewers explained the project and their role and obtained written consent from participants. The interviews were conducted at these community harm reduction sites in confidential spaces, such as a vacant office in the drug user organization and an available nursing room at the harm reduction site. All interested participants were interviewed once (without repeated interviews), with most interviews lasting between 20 and 40 min. Study participants were provided a CAD$20 honorarium and there was no expectation of follow-up to review transcripts, as collecting contact information was not part of the study design. Basic field notes were taken by interviewers, and transcribers also prepared analytical notes, to contribute any notable contextual factors that could inform the analysis. The research team met regularly and reviewed progress and determined when we considered saturation was possible. Ethical approval was obtained from the Health Research Ethics Board at the Island Health Authority (J2018–069).

Critical harm reduction and social justice perspectives and a socioecological model guided our analysis to explore the potential role of drug checking within the overdose crisis. In taking a critical harm reduction and social justice lens to this work, we sought to avoid a neoliberal version of harm reduction that focuses primarily on individual responsibility to abstain from use and potential blame [27, 28], while respecting a basic right to use substances [29] and that people who use substances have rights to services to enable their harm reduction [30]. This perspective views drug checking as a service that recognizes many people use substances for pleasure [31], and resists the institutionalization of this grassroots harm reduction service as a response to the current illicit drug overdose crisis [6].

Socioecological models have been used for considering community health and public health research and action as they are useful for considering the interplay between individuals and their environments, and the impacts of interventions within complex systems and among multiple levels of systems [32–34]. Multilevel socioecological models assume that the action within each level will influence the other levels, and that associated benefits are cumulative [35]. Socioecological models can be used to understand the nature of a public health issue, and to consider development, implementation, and evaluation of public health interventions. Specific levels used within a model range between models, and are often adapted to be most appropriate to the specific research question. Here we used a socioecological model to consider potential impacts of drug checking as a harm reduction intervention at different levels as a way to explore impacts beyond those of individual behaviour change and abstinence goals. Furthermore, we sought the perspective of people who use substances as the basis for constructing these benefits and impacts in line with critical harm reduction and social justice perspectives that emphasize including those with lived experience to ensure research is relevant and respectful.

The principal investigator (BW) and two research associates (TvD & FP) reviewed, coded and compared initial themes that inductively emerged on a small subset of transcripts. Based on this and feedback from community interviewers on any themes that they detected from the interviews, we then developed an initial coding structure to understand potential impacts of drug checking services, from the perspective of prospective service users. Two research associates (TvR & FP) coded all transcripts into this initial structure in NVIVO 11 (QSR International). The Principal Investigator (BW) and research team (TvR, FP, BP, DH) then inductively developed critical themes around potential impacts of drug checking. Based on prior work, we first defined the central action for community-wide drug checking that would confer these potential benefits and impacts: informing on the composition of substances, including what active ingredients, adulterants, and cuts may be present [8]. This includes providing information on the presence of fentanyl or fentanyl analogues (similar to what is provided by fentanyl test strips), other components in a drug sample that may have unexpected effects, and information on concentration and potency where possible. We then sought to understand who might benefit from drug checking, and how these benefits and impacts would operate across multiple levels.

Drawing on sociological approaches, a model was constructed from these themes by grouping resulting impacts across four main levels: the individual level for people who use substances, the market level, the community level, and the public policy level. Within each level, intermediate outcomes such as impact on behaviours or processes were considered as well as the impact on more distal outcomes and influences on other levels. Based on this, we constructed an overarching theme for the impacts at each level of the socioecological model, as well as considered key themes within each level. All authors including those involved in the interview process reviewed the analysis and interpretation.

## Results

Of the 27 people interviewed, about half of the participants identified as men and most were individuals with low-incomes and receiving social assistance. More than half lived in supportive or subsidized housing, single room occupancy or rooming houses, or were currently experiencing homelessness. Most (70%) reported daily substance use, and six participants reported having overdosed in the past 6 months. Characteristics of the sample are given in Table 1.

**Table 1:** Characteristics of the sample (*N* = 27)

Characteristic	Number (Percent)
	n (%)
**Gender**^**a**^	
Man	14 (51.9)
Woman	13 (48.2)
Non-binary/Transgender/Other^d^	0 (0.0)
**Age in years**^**abcd**^	
20–29	4 (22.2)
30–44	6 (33.3)
45 or older	8 (44.4)
**Identified as Indigenous (First Nations, Métis or Inuk)**	
No	23 (85.2)
Yes	4 (14.8)
**Sexual orientation**^**abcd**^	
Heterosexual or straight	14 (82.4)
Lesbian or gay/Two-spirit/Queer/Bisexual/Other/Don’t know	3 (17.6)
**Current level of education**^**abcd**^	
Some secondary education	3 (16.7)
High school diploma or equivalency	5 (27.8)
Apprenticeship, trades certificate or diploma, other certificate, diploma or degree	6 (33.3)
Other	4 (22.2)
**Current living situation**^**acd**^	
Apartment/house	9 (33.3)
Supportive or subsidized housing/Single room occupancy or rooming house	9 (33.3)
Public place, or street	6 (22.2)
Family or friend’s place, other	3 (11.1)
**Primary source of income**^**d**^	
Wage or salary including from own business	4 (14.8)
Disability benefits	16 (59.3)
Welfare or income assistance	4 (14.8)
Other	3 (11.1)
**Personal income last year**^**acd**^	
Less than $20,000	13 (50.0)
$20,000 to less than $40,000	7 (26.9)
$40,000 or more	3 (11.5)
Don’t know	3 (11.5)
**Frequency of illicit substance use**^**d**^	
Daily	19 (70.4)
Three or more times per week	2 (7.4)
Once or twice per week	3 (11.1)
Occasionally (not every month) or Never	3 (11.1)
**Overdose within last six months**^**c**^	
No	20 (76.9)
Yes	6 (23.1)

^a^Does not sum to 100.0% due to rounding

^b^Demographic questions were altered- not asked of 9 participants in early interviews

^c^Does not sum to 27 due to missing data

^d^Some categories were combined due to small numbers or a lack of responses, and/or to preserve anonymity

Participants in this study discussed who might benefit from drug checking services and what they perceived to be the potential benefits (Table 2).

**Table 2:** Selected quotes by themes

Themes	Selected quotes
Who benefits?	“It would work well for anybody, anybody who is using drugs, I think. Women, youth, men, everybody. Everybody who needs it, who needs to know. (INTV F2) “And then you’d get the parents that are really informed that would show up. Like ‘Yeah, I want to know what my kid’s into. So, if he does go down I know what I can do to help him stay alive’” (INTV F9). “Well you definitely want to know what you’re selling. You don’t want to kill anybody. You don’t want to, you know, be selling heroin and have no heroin in it” (INTV B1). “It would be beneficial to me because I sell drugs. Not huge quantities, just to my friends so that they’re safe, in my building, so that they’re safe. This way, it will be an even better, this will make me feel more better about selling my product, because I’m always worried that I’m going to sell pure fentanyl and kill someone. It really upsets me. The reason that I do sell is because a lot of my friends were dying because they were getting pure fentanyl, and I wanted to be one of those that sold a product that helped with their pain, like heroin, and to get high. Not pure fentanyl where you’re not going to wake up. So this way I’d be able to, like if I got a different product, I’d be able to get it tested so that I know the product I have that I’m going to be selling to other people is safe, and that’s wonderful to me” (INTV F1)
What are the potential benefits?	
• Individual level: Drug checking to improve health and wellbeing of people who use substances	“The only way you can really tell is when you’re actually doing it” (INTV B2). Overall, people want to know “if it’s what they’re actually buying and not, um, something a lot more deadlier or something that they’re allergic to that they didn’t intend on buying” (INTV P5). “It’s harm reduction, knowing what you’re putting in your body, injecting in your body. If it’s bad or if it’s going to harm you. It’s going to reduce the harm, yeah. That’s why I get it drug checked, because I don’t want to put …, something that’s not normal, I won’t be using that product.” (INTV F1) You know, if it’s a lot more fentanyl then I’ll end up smoking it more, rather than shooting it, right?” (INTV B3). “I think that it’s bringing in drugs that we’ve brought in from the street to get checked and make sure that they are what they say they are. I know I’ve had really, really bad experiences and gotten what I thought was heroin and it ended up being ketamine and I’d never done that before and it was a horrible, scary trip. So I personally like to get mine checked. You don’t know who it’s coming from. … I’m just too scared that I’m going to get some bad drugs and something really horrible is going to happen.” (INTV F2) “I tried the service a few times and it made me feel a lot more at ease to know that drugs that I was using was what I thought it was for safety and just for my wellbeing, it really helped me a lot” (INTV F1).
• Market level: Drug checking to increase quality control in an unregulated market	“I would know like, where the bad drugs were coming from? Because now it’s random. Right? So it’s like, it’s not consistent. Like, if every time I got drugs, or pretty well close to every time I got drugs, I got them tested, I would be able to find out which person is giving out more fentanyl, or more heroin, or more carfentanil, and then I would stop going to them and start going to like, you know what I mean? I’d be able to figure out which source I’d want to go to right?” (INTV B2) “If I know a product I’ve brought in has got, say, mostly fentanyl in it, I’m not going to be buying that. I won’t be going back to that guy. If it’s mostly some cut that’s garbage, of course I’m not going to be going back. … It’s mostly to find out A) if it’s going to work, if it’s good dope, which is what we all want. The dealers want it, the buyers want it, I think we all want it. We want to know what’s in it because if we can tell people what’s in it, then they will stick to…if I can tell my people, “This is 50% heroin and it’s got some caffeine and this and that,” if I can tell them that, then they’re going to want to keep going through me.” (INTV F1) “In a way, they do have control of some of it because if they didn’t buy off the other guy, let’s say the bigger guy, the bigger block, then the bigger block would get smaller. And if the bigger block gets smaller, then the bigger block has to change their stuff, to stay the same or get bigger. Nobody wants to go smaller. You go smaller and you’re small enough, they wipe you off the planet and you won’t be doing it anymore.” (INTV F6) “I think in a sense it might make people who are dealing the drugs think a little bit more about what they’re putting into the drugs because they know that they could be tested. And people will be pissed if they find out that they spent x amount of dollars for something that only contains half of the active ingredients or, you know, they thought they were getting heroin and then all they got was pure fentanyl. So I think it would change – you know, it holds people who are – which I know people who are processing and selling the drugs don’t have a lot of morals anyways, I think it would at least, they would know they could get caught on the crappy cuts that they’re making at least.” (INTV F17)
• Community level: Drug checking to create healthier environments	“Well what’s going to be good is if you can convince the world that it is a good thing. Like you know, convince people that it’s, it’s not all about junkies, and street homeless people, and bums, and drug addicts. You know what I mean? It’s about everybody. Even a parent who is worried about their child doing drugs. Because I know, I know a parent that, his son is an addict. Right? He’s in his twenties, he’s an adult. But still, his dad, you know, whenever his son comes up dope sick and wants to get some dope, his dad will go get it for him. And his dad’s been using the service in here. Like that’s totally a rare situation, but still. You know, he never would have done that before. But since there is that drug checking and it made him comfortable for him to do that for his son. You know, because he doesn’t want to see his son hurting, but he doesn’t want to give his son money because all the times he’s done that, every time he gives his son money, he’s been picking him up from the hospital for overdosing.” (INTV B3) “it’s an actual moral issue. Most people have not had respect. They’ve been raped, beaten and put down their whole life … if it took one person’s kindness to bring me back, what if they showed one person kindness, how many would come back? It would be frickin’ amazing” (INTV F4).
• Policy level: Drug checking to mediate policies around substance use	“Legalization is a huge part of drugs, that’s why there’s shit in them. So yeah, we need everybody involved in the drug world. The government, everything” (INTV F5). “It’s a human right. A person drinking would expect it, a person having a tea would expect it. A person drinking water would expect it. Why can’t we?” (INTV F4).

### Who benefits?

We heard that there was a shared need for drug checking between three significant (overlapping) groups:

- People who use substances
- People who care for people who use substances
- People who sell substances

People who use substances are extremely diverse as substance use is common across demographic groups, and we heard that having information about the composition was considered a shared need that would benefit any person who uses substances. People who care for people who use substances may choose to test on behalf of family, friends and community members. For example, participants noted the service could have value for parents concerned about overdose. They also indicated that in the case of overdose or death, people might seek to test to find out what was in the substance. Participants also indicated that drug checking could be extremely valuable for people who sell substances and should be made safe and accessible for people who sell in order to protect others. We heard how many people who sell are concerned about the safety of others and want to be as informed as possible about the composition. Overall, we heard that the population who could benefit from community drug checking services would be wide and the resulting benefits would extend beyond a narrowly defined target population that considers those ‘at most risk’. This supports the need for a universal approach, with policies that support delivery of *community-wide* drug checking.

### What are the potential benefits?

**Fig. 1:**
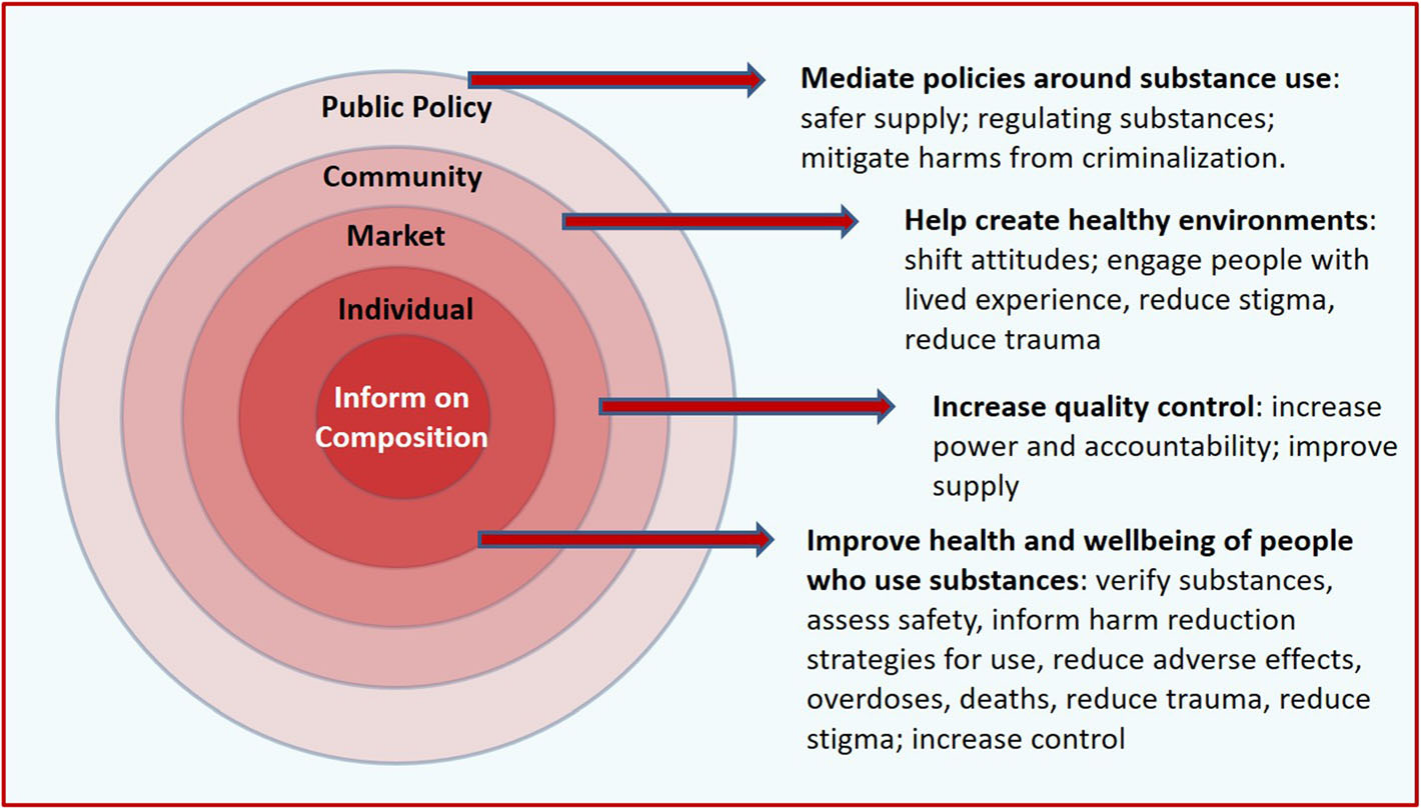
Potential impacts of drug checking services: Informed by perspective of prospective service users

Within a socioecological model consisting of individual, market, community and public policy levels we identified four overarching themes around potential benefits from drug checking (Fig. 1). These included: (1) Drug checking to improve health and wellbeing of people who use substances, (2) Drug checking to increase quality control in an unregulated market, (3) Drug checking to create healthier environments, and (4) Drug checking to mediate policies around substance use.

#### Individual level: drug checking to improve health and wellbeing of people who use substances

We heard that drug checking could operate at the individual level to impact knowledge, personal skills and health outcomes for people who use substances. Participants described a number of potential benefits of drug checking related to the health and wellbeing of people who use substances which included verifying substances and assessing relative safety to inform personal harm reduction strategies, and improving health outcomes. Knowing the composition was considered important regardless of the substance, and it was difficult even for those with a great deal of experience with substances to know what they were buying. Participants also noted that *confirming* that a substance is what they expect would be considered a valuable result. When asked whether drug checking results would change their decision about using a substance, many indicated that it wouldn’t, but that they would still test it, for example, if a substance contained fentanyl: *“it’s just nice to know if it’s in there” (INTV B3).* While many participants had concerns over the presence of fentanyl, this extended to other ingredients that might be deemed harmful or unwanted, for example, *“if it was cut with bath salts, no, I wouldn’t use it” (INTV F11).*

Being informed about the composition enables harm reduction with some participants reporting they would be *“more cautious” (INTV F17)* in their use. When asked whether knowing the composition would affect their use, participants indicated a number of personal harm reduction strategies they might employ that fit with this including: smoking rather than injecting, smoking a bit to start, altering how they mix it, where they use it, or using less. Overall, we heard how increased knowledge about drug composition would provide more information to support decisions for drug consumption and harm reduction strategies.

Participants identified a number of ways that knowing the composition, would improve health outcomes such as reducing adverse effects, overdoses and death, as some simply stated: *“It saves lives” (INTV F4).* In addition to reducing negative health outcomes, participants indicated that verifying the composition could improve mental health and wellbeing by offering reassurance. These findings highlight the lack of control that people who use substances currently have and how drug checking could operate to increase this sense of control.

#### Market level: drug checking to increase quality control in an unregulated market

We heard that drug checking could operate to influence the relationships within the market, through increasing power and accountability, and to improve the quality of the supply. Participants indicated that they had extremely limited power in their interactions within the market. They reported that one of the main strategies for getting quality substances they deemed safe was through trying to buy substances from someone they have had good substances from in the past. However, they noted that this was challenging as many people may also not know what they are selling. Within this context, participants identified that one of the critical benefits of drug checking services would be to shift power and accountability within the market as knowing the composition of a substance would allow them to pick a better source for the future. This could benefit people who sell substances as well, as they could verify the composition of the substances they intend to sell, and increase trust and loyalty with people who are buying. Thus, drug checking would produce a benefit on both sides of the market.

Overall, our findings support that knowing the composition could increase power and accountability within a market that currently has very little, with benefits for both people who buy and sell substances in their interactions. Such increases in power and accountability could create a pressure that result in improved supply which would be a significant impact of drug checking services. Participants identified a number of potential impacts that could result in improved supply including being able to select different sources, demand better products, and testing products for sale. Further, as participants noted, drug checking would allow people who sell substances to also seek out better sources and create pressure higher up in the chain. This pressure could help change the way substances are being cut or mixed to result in improved supply. Overall, this could be one of the more powerful impacts that drug checking is able to make that can help to reduce individual risk for negative outcomes and improve the health of the population.

#### Community level: drug checking to create healthier environments

We heard that drug checking could have impacts at the community level on attitudes, behaviours and health outcomes across the population. We heard that drug checking services could have a role in creating healthier environments by shifting attitudes to increase respectful treatment for people who use substances and reduce stigma; engaging people who use substances; and improving population health by reducing trauma from overdose. It could also have a role in providing information to the community about what is circulating in the supply.

Participants noted that drug checking services could operate to shift attitudes and values to be more accepting and respectful of people who use substances. They noted attitudes had become more accepting towards substance use over time, and drug checking could support this further. For example, one participant noted that drug checking could further support changing attitudes to allow more people to access harm reduction services. Another participant reported that the most significant impact of drug checking would be increased respect and dignity for people who use substances. They noted the poor treatment and high levels of stigma that people who use substances face, that compounds the violence and trauma they may already have experienced. These shifting values and attitudes that reduce stigma around substance use would help create environments that support better health and wellbeing overall.

Further, another participant noted that services can provide social support and a sense of community, and that where people who use substances are included in the operation of the service, this would allow people to” *fit in and feel productive” (INTV F5).* This would contribute to the person’s well being, as well as healthier communities. Overall, we were told if drug checking were able to shift the supply so it is safer and reduce negative health outcomes, this would also support healthier communities, with less trauma for members of the community from overdoses and deaths.

#### Policy level: drug checking to mediate policies around substance use

We heard that drug checking could influence public policies around substance use. We identified that at the policy level, drug checking could have a role in supporting safer supply initiatives and regulation of substances to ensure relative quality and safety, and inform drug policy. We also heard that it could be used to help protect people who use or sell substances by establishing evidence to mitigate harms of criminalization. Some participants discussed the impact of criminalisation and prohibition on the supply, with it being the reason that so many substances are relatively unsafe. One participant noted that the toxic drug supply is due to criminalisation and that government engagement and policies are needed to address this.

Further, participants indicated that a role of drug checking should be to support safer supply as was identified at the market level. For example, one participant noted that drug checking should be “*mandatory” (INTV F4)* for anyone selling substances as part of a move towards creating a safer supply. Further, they stated that knowledge about what is in a substance should be available to all involved, including people who sell and use substances, as this knowledge is a human right. This aligns with the idea that drug checking could have a role in regulation of substances and safer supply, a potential impact of drug checking could be to increase safer supply through testing, as well as show the need for safer supply initiatives and policies to regulate substances to protect people’s health.

## Discussion

This study explores the potential role of community drug checking services within the context of the overdose crisis, from the perspective of people who use substances and other prospective service users. We sought to expand the focus of drug checking beyond that of an individual level intervention, by adopting a socioecological model to identify potential impacts at multiple levels.

Our findings support a shared need for drug checking that requires a universal approach. Moreover, these findings support a potential role for drug checking as a meaningful harm reduction response that should not be conceptualized only as an individual level intervention with a behavioural change mandate, and should consider the potential influence at other levels such as the market, community and policy levels as identified here. There is potential interest from people who sell substances to access drug checking while the criminalization and stigmatization of substances and people who sell substances creates challenges in achieving these potential benefits [8]. Kolla and Strike have described the potential to integrate people who sell substances in harm reduction programming, specifically in addressing the impacts of fentanyl in the unregulated drug market [36]. Bardwell et al. [24] recommend community drug checking programs consider ways to engage people who sell substances as their customers place a high level of trust in them to identify and communicate drug contents. Trust, rapport, engaging people with lived experience and rapport building are consistently noted as essential to respond to the context of criminalization and stigmatization and these are perhaps heightened for people who sell substances [8, 10, 36, 37].

At the individual level, participants described community drug checking as an intervention to inform use and personal harm reduction practices, not to result in disposal of substances or abstaining from use. Community drug checking was valued for quality assurance and confirmatory checking of illicit substances, not only to detect potentially dangerous or novel ingredients. The perception that drug checking functions solely to alert people of the dangers of adulterants and other unexpected ingredients is limited, as we heard there was value in providing results that confirmed ones’ purchase and potential quality of this.

Community drug checking has potential impacts at the market level and functions for both people who buy and sell substances. For those buying substances, the service can inform purchases, and influence which sources are more valued and which less dependable or consistent. With the illicit drug market frequently described as toxic and unpredictable, notably with the introduction of fentanyl, community drug checking can also benefit people who sell substances. As a potential market-level monitoring function, drug checking utilizes consumer-derived data directly from people who use substances to generate drug market reporting that is potentially more reflective of the market than police seizure or forensic data [38]. Brunt et al. [38] is one advocate for drug checking data being utilized at a regional level for its potential to influence drug manufacturers and people who sell substances as purchasers test their product and have greater control over their purchases and the market. Drug checking therefore is seen as holding a quality control function in the unregulated market that is consumer generated and trusted as a ground up rather than top-down measure from law enforcement or others [39].

At this market level, drug checking may have a monitoring and reporting function that could inform buying and selling and ultimately increase overall drug-quality resulting in a safer supply as we identified here [7, 23]. The Drug Information and Monitoring System (DIMS) is one early example funded by the Ministry of Health in the Netherlands [40]. The Trans European Drug Information (TEDI) project is an example of a central database for multiple European drug checking projects for analysis and monitoring [38]. Drug alerts, warnings and bad dope reports are a potential product and service from drug checking at this meso level. As a public health surveillance tool, drug checking can often convey results in the context of risks and messages of non-use or avoidance. Because drug alert messaging to promote behavioral change possibly holds limited effectiveness and potential unintended actions [41], there are recommendations for more user-informed messaging of greater relevance [42]. Gine et al. [39] for example advocate for a harm reduction approach to warnings and alerts as an alternative to the scare tactics of governmental and law enforcement reports that promote abstention. This harm reduction approach is described as drug user derived data that is more trustworthy and individually-tailored to be more pragmatic and potentially more rapid and relevant.

At the community level, many of the impacts already identified contribute to an overall healthier community as the benefits are reciprocal and cumulative, and the overdose crisis impacts communities, not just individuals. For example, if drug checking can contribute to reduced overdose and deaths for individuals who use substances, this also protects communities from associated trauma and grief. Further, as participants noted, drug checking may have a role in shifting attitudes around substance use, in part through services that are perceived as sanctioned and through any resulting policy changes. Increasing respectful treatment of people who use substances, and reducing stigma and trauma improves the health of communities. This also makes drug checking more accessible for those who may not have previously been comfortable doing so, including for people who use or sell substances, or test for others in the community.

Finally, at the public policy level, as public health increasingly explores decriminalization and safer drug supply initiatives [43, 44], such as heroin buyer’s clubs [45, 46], the role of community drug checking could be explored within these programs and policy shifts. The sanctioning of community drug checking within safer supply purchasing and distribution could enable a non-medical model of safer supply that continues to source from the current drug markets but with quality assurance practices and reporting. If community drug checking is integrated within public health it has the potential outcome of long-term public health monitoring and reporting and ultimately for more informed drug policy implementation [7, 23].

Our findings confirm that as a critical harm reduction response, drug checking must center people who use substances and the ways in which the programs and services best meet these needs and priorities. Integral to critical harm reduction is also drug checking’s potential to address the systemic causes of drug risks and harms such as criminalization, stigmatization, structural violence and inequities among other interrelated social determinants. Emulating a central harm reduction principle of “meeting people where they are at”, drug checking can accept the illicit drug market where it is at and engage with the market and people who sell substances to provide pragmatic information and interventions. These are important benefits for the shorter-term to improve quality assurance and reduce risks and harms related to criminalization and stigmatization in the absence of structural policy change such as decriminalization and a more accessible safer supply. The findings resemble the position of Andrew Groves’ pragmatic analysis of festival pill checking which he asserts cannot eliminate the harms of substance use nor is it intended to [7]. Rather, both community drug checking and festival pill checking programs should function within larger harm reduction strategies as a non-punitive, pragmatic response to drug use [7].

The concrete impacts identified here at multiple levels offer insight into more appropriate evaluation for drug checking. Our findings highlight a range of potential measures including whether service users: obtained information they deemed useful to inform use and harm reduction strategies; felt that drug checking improved their overall health and reduced their risk of adverse effects, overdose and death; provided information that informed choice and control within the drug market, or the ability to offer products to others that are safer and higher quality; improved stigma and attitudes around substance use within their communities. Appropriate evaluation may also examine community attitudes and experiences around drug checking, changes in the supply over time, as well as changes in drug policy. Finally, evaluation of drug checking at these multiple levels can ask not only what works, but also what works for whom and in what circumstances, to be most responsive to the diversity of substances, people who use substances and contextual factors that impact both service delivery as well as service uptake and effectiveness. An explicit health equity lens to evaluate drug checking should be considered to highlight system level barriers and inform tailoring of services to best respond to the unique needs of groups and individuals.

### Limitations of the study

This study consists of both strengths and limitations. A strength of the study is that it seeks the perspectives of people who use substances to understand the potential impacts of drug checking services within the context of the overdose crisis. The study was based in Victoria, BC, Canada where the burden of the overdose crisis is extremely high. Our collaborative research team included community partners from the local harm reduction and drug user organizations. We used a socioecological model to aid in identifying impacts beyond those privileging individual behavioural change toward abstinence. We sought to identify benefits from the perspective of people who use substances in line with principles of harm reduction and social justice to understand what is most relevant to people most impacted by the overdose crisis. However, as many research participants had little knowledge of or experience with drug checking this may have limited their insight into how drug checking could potentially be beneficial. Moreover, interviews did not specifically query what the potential benefits might be at different levels. That data was sparser for the community and policy level likely reflects both of these factors. Recruitment within harm reduction sites would have limited our reach to populations not accessing these services who could potentially benefit from drug checking services. Future research on community drug checking is needed to explore the perspectives of youth and young adults, people with higher incomes, suburban and rural populations, Indigenous peoples and people of colour, women, and those identifying as queer, trans and non-binary, among others. Our research indicated that drug checking services may be used by people who test for others or to sell, future research is needed to explore perspectives of such third party testers.

## Conclusion

Community drug checking is emerging as part of an integrated response to the overdose crisis that requires a universal approach to reach the diverse population that could benefit from this. Overall, community drug checking is extremely limited and less responsive to the community if its dominant focus is on individual behavioural change, that prioritizes alerting people of potential danger and measures success as disposal or non-use of substances. Community drug checking may operate as a meaningful harm reduction response with impacts at and beyond the individual level. These include increasing power and accountability within the illicit drug market, improving the health of communities, and supporting safer supply initiatives and regulation of substances at the policy level. These benefits are restricted by the contexts of criminalization and stigmatization, and the integration of community drug checking within public health and harm reduction is vital to realizing these outcomes. While more limited, participants highlighted drug checking as one way to mitigate harms from criminalization and mediate abstinence-based drug policies. Evaluation of community-wide drug checking interventions need to reflect the potential impacts identified here that extend beyond the level of individual behaviour change and recognize lived realities and structural conditions.

## Supplementary Information

**Additional file 1.** Implementing Innovations in Drug Checking: A Harm Reduction Pilot in Response to Illicit Drug Overdose. Pre-Implementation Interview Guide. Additional file containing the qualitative interview guide.

**Additional file 2.** Consolidated criteria for reporting qualitative studies (COREQ): 32-item checklist.

## Data Availability

Data are not available due to the sensitive, confidential and potentially incriminating nature of the data.

## Abbreviations

BC: British Columbia
DMIS: The Drug Information and Monitoring System
TEDI: The Trans European Drug Information
