# Supplementary material for: The potential impacts of community drug checking within the overdose crisis: qualitative study exploring the perspective of prospective service users
Source: BMC Public Health. 2021 Jun 16;21:1156. doi: 10.1186/s12889-021-11243-4 (PMC8207696; doi:10.1186/s12889-021-11243-4)
Supplement: Supplementary file 1 — Additional file 1. Implementing Innovations in Drug Checking: A Harm Reduction Pilot in Response to Illicit Drug Overdose. Pre-Implementation Interview Guide. Additional file containing the qualitative interview guide. [file 12889_2021_11243_MOESM1_ESM.pdf]

Implementing Innovations in Drug Checking: A Harm Reduction Pilot in Response to Illicit Drug Overdose.

Pre-Implementation Interview Guide

Semi-structured interview guide with potential prompts

1. Can you tell me what your contact/involvement has been with the [harm reduction] agency? (Prompt: How long have you been coming here? Why do you come here?)

2. This interview is to ask people about their thoughts about drug checking, so maybe a good question to start with is to ask you how you describe drug checking as a service? How would you describe drug checking to someone who has never heard of it? (Prompt: Do you have previous experience with drug checking?) What do you think about drug checking?

3. What would you hope for in a drug checking service? What would your expectations for a service be? What would be the most significant impacts of a service? (Prompt: If you could design a perfect service, how would it operate?)

4. How would a service fail to meet your expectations? Can you give some concrete examples? (Prompt: What would make you never want to come to a drug checking service? Or never come back to one?)

5. What is it about this location that could make a difference to how drug checking would work (or wouldn't work well?) How could this service work differently in different places?

6. Would a service change your action around how you use drugs, purchase drugs, or sell them? (Prompt: Could you give an example?)

7. What matters about how the service is done, in order for it to be a good program? Can you think of some things that are barriers to this service working well for you? What about any facilitators that help?

8. What about the people involved in a drug checking service – the people who develop it and the people who provide the service. Can you tell me how the individuals involved could change you accessing or benefiting from the service?

9. There are lots of ideas of how drug checking could work or how it may not work and how it may work well for some people but not others. Who would a service work well for and who would it not work well for and why? In what ways (would it work or not work well for different folks)?

10. Have you heard of the term ‘harm reduction’? (Prompt: One definition of harm reduction is a set of practical strategies and ideas aimed at reducing negative consequences associated with drug use. Harm reduction is also a movement for social justice built on a belief in, and respect for, the rights of people who use drugs). Do you think this agency is providing harm reduction and follows harm reduction principles? Can you give an example?

11. As one of our final questions, we are wondering about how the larger context may impact a drug checking service and how it can work well or not. When you think about the overall context – outside of these walls - what do you think impacts how effective a drug checking service is for you? Can you think of some things that are barriers to this service working well for you? What about any facilitators that help? (Prompt: How useful is drug checking in the contexts of criminalization, poverty, or the stigma surrounding many types of drug use?)

12. What needs to be considered when implementing services such as this? What should be the process for implementing drug checking?

13. What else do you think we need to know, to really understand how a service would work here? (Prompt: Is there anything else we haven’t covered that you want to get across?)
